# Development of GABARAP family protein-sensitive LIR-based probes for neuronal autophagy

**DOI:** 10.1186/s13041-019-0458-z

**Published:** 2019-04-08

**Authors:** Pureum Jeon, Ju-Hui Park, Yong-Woo Jun, You-Kyung Lee, Deok-Jin Jang, Jin-A Lee

**Affiliations:** 1 Department of Biological Sciences and Biotechnology, College of Life Sciences and Nanotechnology, Hannam University, 461-6 Jeonmin-dong, Yuseong-gu, Daejeon, 34054 Republic of Korea; 2 Department of Ecological Science, College of Ecology and Environment, Kyungpook National University, 386, Gajang-dong, Sangju-si, Kyungbuk, 37224 South Korea

**Keywords:** LC3, GABARAP, LIR motif, Autophagy

## Abstract

Autophagy allows for lysosomal cellular degradation of cytosolic components. In particular, neuronal autophagy is essential for cellular homeostasis and neuronal survival and is tightly regulated by several autophagy-related (ATG) proteins in post-mitotic neurons. Among these ATG proteins, the LC3/GABARAP proteins are known to regulate autophagosome biogenesis/maturation and cargo recognition. However, little is known about the role of GABARAP family proteins in neuronal autophagy despite their abundant expression in post-mitotic neurons. We have previously developed HyD (Hydrophobic Domain)-LIR (LC3-interacting region)-based autophagosome markers. In this study, to monitor GABARAP family proteins in autophagosomes of post-mitotic neurons, we improved the sensitivity of the probes for specifically detecting endogenous GABARAP family proteins by adding one more LIR motif to the LIR probes. We have tested the efficiency of two different LIRs, from ULK2 and Stbd1, in regard to their cellular localization to autophagosomes. HyD-2xLIR(ULK2)-GFP and HyD-2xLIR(Stbd1)-GFP demonstrated specific localization to GABARAP-positive autophagosomes relative to LC3B-positive autophagosomes in MEF/HeLa cells in an autophagy-dependent manner. Indeed, HyD-2xLIR(Stbd1)-GFP could efficiently detect GABARAP-positive autophagosomes in cultured cortical neurons. Our improved GABARAP-sensitive probes will contribute toward understanding the specific role of GABARAP family proteins in regard to neuronal autophagy.

## Main text

Autophagy is a highly regulated cellular pathway involved in lysosomal degradation of unnecessary and/or dysfunctional cellular components wrapped by a double-membrane–bound autophagosome within the cells [1]. In a highly polarized neuron, neuronal autophagy is essential for cellular homeostasis and cell survival under physiological and pathological conditions due to its post-mitotic nature and dynamic signaling at the synapse [2]. Among the many ATG (AuTophagy-related Gene) genes, the ATG8 protein is involved in autophagosome formation, cargo recognition and recruitment to autophagosomes. In human cells, at least seven ATG8 homologues (mammalian ATG8, mATG8) are expressed. These are generally divided into the two subfamilies of GABARAP proteins, including GABARAP/−L1/−L2 as well as LC3, which includes LC3A(a,b)/B/C. Interestingly, the GABARAP family proteins, relative to the LC3 proteins, are localized to the initial segment of axons as well as being present in synaptic-vesicle–enriched fractions [3]. However, the differential roles of each LC3/GABARAP family protein in neuronal autophagy or selective autophagy are largely unknown. In addition, the ability to detect LC3/GABARAP proteins in polarized neurons is limited, as overexpression of LC3 family proteins can cause abnormal branching or induced protein aggregation in an autophagy independent manner [4].

**Fig. 1:**
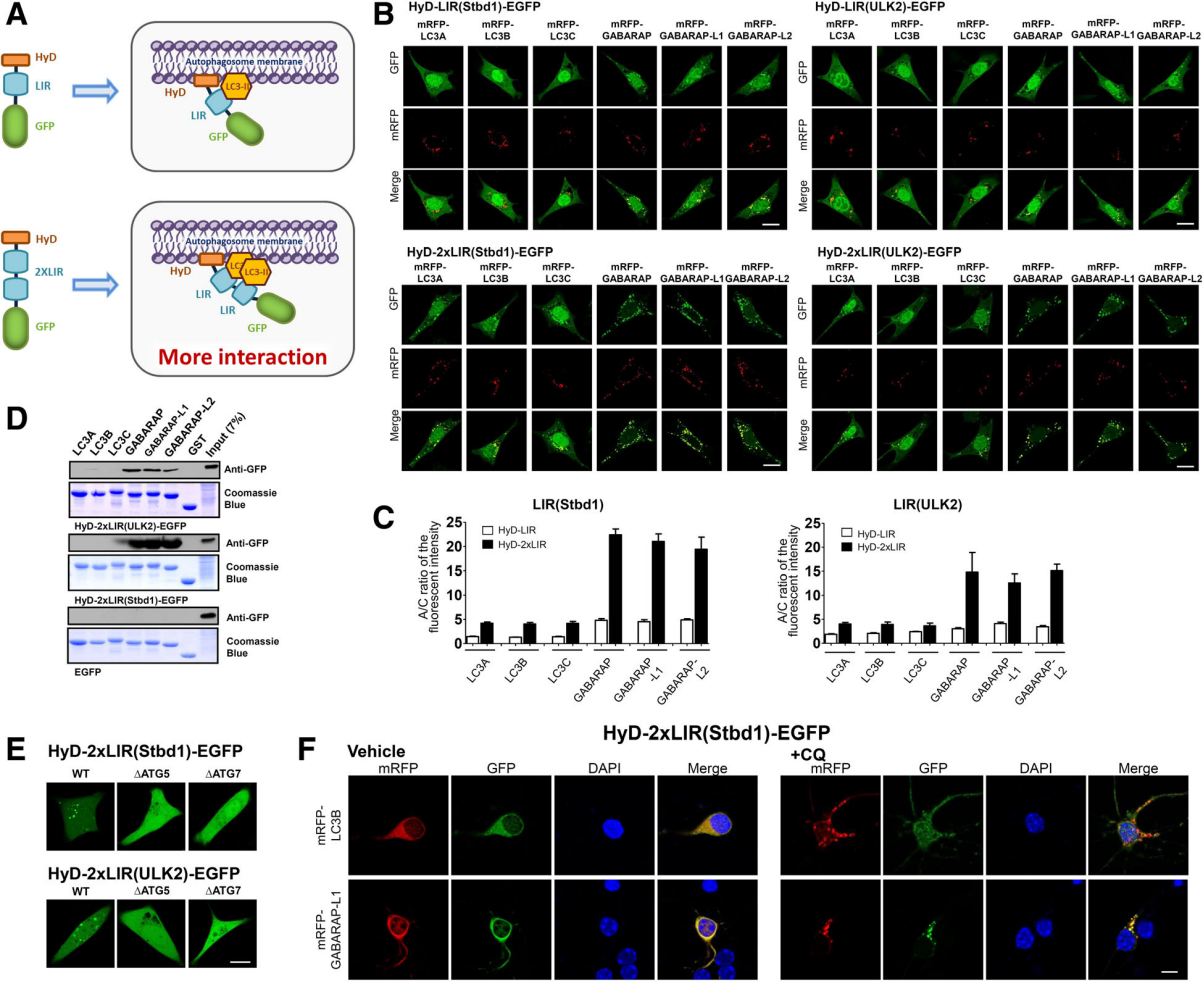
Efficient localization of HyD-2xLIRs-GFP to mRFP-LC3/GABARAP-positive autophagosomes. **a** Schematic model depicting the development of new autophagosome sensors. LIR: LC3-interacting region, HyD: hydrophobic domain. **b** Confocal images showing the cellular localization of HyD-LIR(X)-GFP or HyD-2xLIR(X)-GFP together with mRFP-LC3A/B/C and GABARAP/−L1-L2 in MEFs incubated with 100-nM rapamycin (Rapa) + 10-mM NH_4_Cl for 4 h. Scale bar: 10 μm. **c** The bar graphs illustrate the ratio of autophagosomal/cytosol (A/C) GFP fluorescence intensity in cells expressing HyD-LIR(X)-GFP or HyD-2xLIR(X)-GFP and each mRFP-LC3/GABARAP protein. **d** The LC3/GABARAP protein binding properties of HyD-2xLIR(Stbd1)-GFP or HyD-2xLIR(ULK2)-GFP assessed using GST pull-down assays. **e** Confocal images showing cellular localization of HyD-2xLIR(Stbd1)-GFP or HyD-2xLIR(ULK2)-GFP in wild-type (WT), ATG5-knockout (ΔATG5), or ATG7-knockout (ΔATG7) HeLa cells treated with 100-nM rapamycin (Rapa) + NH_4_Cl. Scale bar: 10 μm. **f** Confocal images showing cellular localization of HyD-2xLIR(Stbd1)-GFP together with mRFP-LC3B or mRFP-GABARAP-L1 in cultured cortical neurons incubated in the absence or presence of 50 μM chloroquine (CQ) for 1 h. Scale bar: 10 μm. X; Stbd1 or ULK2

To overcome these issues, we have recently developed new autophagosome sensors to detect endogenous LC3/GABARAP proteins in autophagosomes using LIR (LC3-interacting region) motifs and a hydrophobic motif (HyD) [4]. In our previous study, among the LIRs identified from different LIR-containing proteins, some of these domains demonstrated selective GABARAP family binding, but these were weakly associate with GABARAP-positive autophagosomes. Therefore, to improve the efficiency of these HyD-LIR-GFP sensors for monitoring GABARAP proteins, we generated HyD-2xLIR-GFP sensors by duplicating the LIR motifs from selective GABARAP family binding proteins, including LIR motifs from the ULK2 and Stbd1 proteins, into HyD-GFP (Fig. 1a).

To compare the efficiency of HyD-LIR-GFP and HyD-2xLIR-GFP in regard to cellular localization to LC3/GABARAP-positive autophagosomes, we transfected HyD-1xLIR-GFP or HyD-2xLIR-GFP together with each mRFP-LC3/GABARAP family protein into MEF cells incubated with rapamycin (100 nM, 4 h), a general autophagy inducer, under inhibition of lysosomal degradation (10 mM NH_4_Cl, 4 h). As shown in Fig. 1b and c, the HyD-2xLIR-GFP sensors could more efficiently detect GABARAP-positive autophagosomes when compared to HyD-LIR-GFP, indicating that the duplication of the LIR motif from ULK2 or Stbd1 could generally enhance its cellular localization to GABARAP-positive autophagosomes. These results indicated that either HyD-2xLIR(ULK2)-GFP or HyD-2xLIR(Stbd1)-GFP could be used as sensors for detecting GABARAP-positive autophagosomes.

To further validate the binding preference of each HyD-2xLIR-GFP marker, we performed GST pull-down assays with each GST-LC3/GABARAP protein. As shown in Fig. 1d, HyD-2xLIR(ULK2)-GFP and HyD-2xLIR(Stbd1)-GFP each bound selectively to GST-GABARAP/−L1-L2, which was consistent with the efficacy of its cellular targeting to GABARAP-positive autophagosomes.

We also overexpressed HyD-2xLIR(ULK2)-GFP or HyD-2xLIR(Stbd1)-GFP in ATG5 and ATG7-knockout HeLa cells. Both HyD-2xLIR(ULK2)-GFP and HyD-2xLIR(Stbd1)-GFP could detect vesicle structures in wild-type HeLa cells but not in ATG5 or ATG7-knockout HeLa cells (Fig. 1e), suggesting that our improved probes specifically detected endogenous GABARAP-positive autophagosomes in living cells.

Finally, we tested whether our improved LIR-based sensors could detect neuronal autophagosomes in neurons expressing mRFP-LC3B or mRFP-GABARAP-L1. To accomplish this, HyD-2xLIR(Stbd1)-GFP together with either mRFP-LC3B or mRFP-GABARAP were transfected into cortical neurons. Indeed, HyD-2xLIR(Stbd1)-GFP localized to mRFP-GABARAP-L1-positive autophagosomes but not to mRFP-LC3B-positive autophagosomes in the presence of a lysosomal inhibitor (Fig. 1f). These results suggested that HyD-2xLIR(Stbd1)-GFP functions as a useful marker for monitoring GABARAP-positive autophagosomes in neurons.

To develop a fluorescence-based LC3/GABARAP-specific sensor, our group and Dr. Ivan’s group have generated LIR-based autophagosome sensors [4, 5]. Our group used HyD to enhance membrane association while Ivan‘s group used a membrane recruitment (FYVE) or oligomerization (PB1) domain. Specifically, in neurons expressing aggregate-prone proteins, overexpressed LC3 or GABARAP might associate with aggregate-prone proteins in an autophagy-independent manner [6, 7]. Therefore, the specific HyD-LIR sensors detailed in this report will be highly useful for the study of autophagy based on their ability to monitor endogenous LC3- or GABARAP-positive autophagosomes.

## Abbreviations

ATG: AuTophagy-related Gene
GABARAP: Gamma-aminobutyric acid receptor-associated protein
HyD: Hydrophobic domain
LIR: LC3-interacting region
